# Effects of digitalized university curriculum-associated teaching on the equilibrium of autonomic neurophysiology and disposition of learners in medical school (EDUCATE-AND-LEARN): protocol for a randomized crossover study

**DOI:** 10.1080/07853890.2021.1996626

**Published:** 2021-11-02

**Authors:** Warunya Woranush, Annahita Sedghi, Mats Leif Moskopp, Julia Japtok, Christian G. Ziegler, Jessica Barlinn, Lutz Mirow, Thomas Noll, Timo Siepmann

**Affiliations:** a Department of Neurology, University Hospital Carl Gustav Carus, Technische Universität Dresden, Dresden, Germany; b Institute of Physiology, Medical Faculty Carl Gustav Carus, Technische Universität Dresden, Dresden, Germany; c Vivantes Klinikum im Friedrichshain, Charité Academic Teaching Hospital, Berlin, Germany; d Department of Internal Medicine III, Technische Universität Dresden, University Hospital Carl Gustav Carus, Dresden, Germany; e Department of General and Visceral Surgery, Medical Campus Chemnitz of the TU Dresden, Chemnitz, Germany

**Keywords:** Autonomic, learning, videoconference, emotions, interactive seminar

## Abstract

**Background:**

Homoeostasis of the autonomic nervous system (ANS) contributes to cognitive functional integrity in learners and can be greatly influenced by emotions and stress. While moderate stress can enhance learning and memory processes, long-term stress compromises learning performance in a face-to-face classroom environment. Integrative online learning and communication tools were shown to be beneficial for visualization and comprehension but their effects on the ANS are poorly understood. We aim to assess the effects of video conference-supported live lectures compared to on-site classroom teaching on autonomic functions and their association with learning performance.

**Methods and Design:**

Fifty mentally and physically healthy medical students will be enrolled in a randomized two-period crossover study. Subjects will attend a seminar, which is held in face-to-face and simultaneously transmitted *via* videoconference. Subjects will be allocated in two arms in a randomized sequence determining the order in which both seminar settings will be attended. At baseline and throughout the interactive seminar subjects will undergo detailed autonomic testing comprising neurocardiac (heart rate variability), sudomotor (sympathetic skin response), neurovascular (laser Doppler flowmetry) and pupillomotor (pupillography) function. Furthermore, learning progress will be evaluated using pre- and post-tests on the seminar subject and emotions will be assessed using profile of mood state (POMS) questionnaire.

**Statistical Analysis:**

Carryover effects will be handled using a two-way repeated measures (mixed model). Between-group differences (baseline vs face-to-face vs videoconference) will be determined using one-way analysis of variance ANOVA followed by Student–Newman–Keul test.

**Limitations and Strengths:**

This study may elucidate complex interactions between autonomic and emotional dynamics during conventional on-site and video conference-based teaching, thus providing a basis for customized learning and teaching methods. Understanding and utilizing advanced distance learning strategies is particularly important during the current pandemic, which has been limiting on-site teaching dramatically in nearly all countries of the world.

## Introduction

Over the last few decades, multiple digital learning strategies have been designed and are now an essential component of modern curricula. Even before the COVID-19 pandemic has restricted on-site education to a dramatic extent, a growing number of educational institutions offered interactive online lectures and multimodal distance learning programmes. After the outbreak of the pandemic, e-learning, video-conferencing and integrative online learning strategies replaced conventional classroom education entirely in multiple programmes across all disciplines around the globe [1,2]. However, effectiveness of these digital learning techniques as well as their impact on psychophysiology is poorly understood and previous research has shown heterogeneous and partially conflicting results.

This study focuses on the physiological reactions that occur during learning. These reactions are largely mediated by the autonomic nervous system and are closely related to emotions. The theoretical principles for emotional processes in learning is commonly summarized by the Control-Value theory of the achievement emotions, which forms a basis for primarily psychological studies [3]. While both autonomic and emotional changes are relevant to the complex mechanisms of learning and need to be viewed in conjunction with each other, autonomic function assessment allows quantitative evaluation of physiological responses.

In addition, the effects of teaching through the screen in real time are possibly subject to filter effects, such as restricted opportunities for subtle communication and a limited sense of community and competition [4]. These limited degrees of freedom in psychosocial interaction might negatively affect emotions during teaching sessions and thereby alter learning success [5]. Emotions in a traditional classroom are considered to be a dominating factor influencing learning success and are modulated by the classroom atmosphere and surrounding, including social relationship between instructor and classmate as well as learning content and learning methods. While positive emotions in a classroom such as enjoyment, excitement, hope and pride may trigger student’s attention and motivation facilitating the learning process, negative emotions such as anxiety, anger, boredom and shame have an opposite effect and can compromise learning progress substantially [6,7].

Whereas research has shown that mild stress may enhance learning, severe and chronic distress negatively affects learning performance and memory recall [8]. Emotional imbalance and transition from mild positive sympathetic activation to long-term stress can be triggered by subtle and complex aspects in the learning environment. Therefore, understanding the individual effects of different learning forms on emotional and autonomic psychophysiological systems might be crucial in designing effective learning strategies, especially in times of the COVID-19 pandemic, which itself has a profound impact on physical and mental health.

Research suggested that web-based interactive learning may improve students' attention, understanding and visualisation [8]. Conversely, recent studies reported that virtual classroom or distance learning during the pandemic increase anxiety and stress levels by isolation, potentially culminating in depression and serious mental disturbances [9,10]. Relaxation, engagement or stress-affected autonomic functions differently during e-learning [11]. Evaluation of functions of the autonomic nervous system were utilized previously to explore the relationship of learning and emotions. In particular, assessment of autonomic sudomotor function *via* sympathetic skin response (SSR) was used to correlate sympathetic activity with the emotional arousal of students working on online courses [12]. In contrast to interactive student–teacher communication, passive learning in self-study does not seem to affect the autonomic nervous system [13]. Furthermore, visually interactive laboratory work was associated with elevated systolic blood pressure and showed tendencies to altered autonomic neurocardiac and sudomotor function when compared to a face-to-face lecture [14].

Taken together, the complex interaction between emotions, attention and autonomic functions in the context of digital learning strategies as well as traditional classroom-based approaches are poorly understood. Closing this research gap might help facilitate the designing of effective and psychophysiologically healthy learning strategies in times of a global digital transformation of education that has been accelerated substantially by the COVID-19 pandemic.

We aim to characterize the effects of traditional on-site versus videoconference-based education on the autonomic nervous system in medical students and to determine whether changes in autonomic functions during the two seminar forms translate into differences in learning success and stress levels.

## Subjects and methods

### Study subjects

We will include physically and mentally healthy medical students in the first three years of their six-year medical degree programme. Study subjects with a history of cardiovascular or neurological diseases as well as those that are currently under any medical treatment will be excluded from the study. Furthermore, subjects with addiction to drugs or alcohol, smokers and pregnant subjects will be excluded.

### Recruitment

This research project will be advertised to recruit medical students from both medical campuses of Technical University of Dresden: Medical Faculty Carl Gustav Carus in Dresden Germany and Medical Campus Chemnitz at Klinikum Chemnitz gGmbH in Chemnitz, Germany. Student representatives and lecturers will be informed about the rational and study plan of the project. Students who are interested in participating will be contacted by the team of researchers.

### Informed consent

Eligible subjects will be informed in detail about the study protocol. Oral and written informed consent will be obtained from each subject. Subjects will be further instructed to have sufficient night sleep, while caffeine and nicotine consumptions are not permitted within three days prior to measurements.

### Study protocol

Subjects will complete a questionnaire that contains six standard demographic questions and ten questions regarding their medical history. Each subject will be randomized to participate in two learning methods of a preclinical seminar within the same topic, in the order of face-to-face and then videoconference or vice versa under open-label conditions. For the study measurements, there will be two subjects and two investigators in every seminar. One investigator will monitor one study participant for the whole period of the measurement. One participant will start with the traditional face-to-face learning, while the other participant will start with video conference method. Regarding the randomization, we will be using tossing a coin method as a simple randomization. The coin will be tossed by the researcher and the participant will guess the result as “head” or “tail”. Heterogeneity of population will be subsided since we use each participant as its own control by applying a randomized crossover sequence of experimental seminar phases. During the learning session, the subjects will interact only with the instructor. The investigators will interact with the subjects only to attach the electrodes and transducer and give signal when to change learning settings. Throughout the learning sessions, the participant will interact with the same instructor within the same learning topic. Repeating students will not be enrolled in this study. The student who already joined the study measurement will not be included for another measurement.

**Figure 1.**
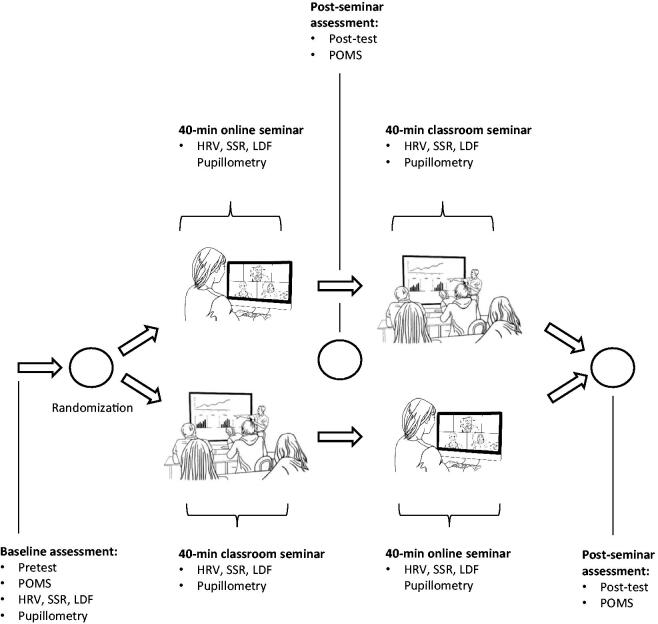
Study Flow. Each subject will be randomized into two types of seminars within the same topic. The autonomic function measurements, topic knowledge, and emotions will be assessed at baseline. Each seminar session lasts 40 minutes. Autonomic functions (sympathetic, parasympathetic nervous system) will be assessed and measured at baseline as well as throughout both types of seminars using heart rate variability (HRV), sympathetic skin response (SSR), laser Doppler flow (LDF), and pupillometry. Emotions and knowledge’s related topic test will be given out as Profile of Mood States (POMS) as well as pre- and post-test.

All measurements will be performed in a temperature and humidity-controlled environment. Measurement time per subject will last around 100 min. This includes measurement at baseline, during seminar settings, and a 10-minute break between settings. The measurements will take place at the same time of day. The study flow is depicted in Figure 1.

All subjects will be evaluated for autonomic neurocardiac, sudomotor, neurovascular and pupillomotor function as well as emotions and learning progress. Before start of a seminar and baseline measurements, a pre-test covering the content of the seminar as well as the Profile of Mood States (POMS) evaluation will be performed to assess knowledge and emotions [15]. The POMS questionnaire is a valid and widely used tool in clinical psychology to determine emotional status by measuring different dimensions of mood swings over a period of time. Subsequently, autonomic baseline measurements will be performed in which the subject will be laying comfortably in a chair with an open angel of 135 degrees. To assess neurocardiac autonomic function, a three-channel electrocardiogram (ECG) will be attached to the chest recording heart rate variability (HRV) as previously described and detailed in chapter “Neurocardiac autonomic function assessment: heart rate variability” [16]. An sympathetic skin response (SSR) electrode will be placed on the index and middle finger to assess sudomotor autonomic function, whereas an Laser Doppler Flowmetry (LDF) electrode will be placed on the ring finger to assess neurovascular function. as previously described and detailed in chapters “Sudomotor autonomic function assessment: sympathetic skin response (SSR)” and “Laser Doppler Flowmetry (LDF)” [17,18]. All electrodes will be attached to the non-dominant hand. Mobile pupillometry will be used to assess changes of pupil size and velocity. The baseline measurements of autonomic functions will last 240 s and will be undertaken after resting for 180 s prior to the start of the seminar. During each seminar session, HRV, SSR, LDF and pupillometry will be simultaneously recorded. After each interaction with the instructor, the subject will perform three deep inspirations. These breathing patterns allow separate assessment of immediate sympathetic response stimulated by deep inspiration as well as parasympathetic stimulation following metronomic breathing. All amplified and artefact-cleaned neurocardiac, sudomotor and neurovascular and signals as well as breath excursions will be converted to digital data using four-channel digitization hardware (Power-Lab 4/26®, ADInstruments, Castle Hill, Australia). The raw data will be displayed using an analysis software (LabChart Pro® 5, ADInstruments, Castle Hill, Australia). During the 10-min break between two sessions, the subject will be instructed to maintain a resting state avoiding any interaction with other classmates and the instructor. After the end of each seminar session, the subject will complete a post-test and another POMS questionnaire. Details on each assessment technique are given in the following.

### Neurocardiac autonomic function assessment: Heart rate variability

Analysis of HRV will be undertaken to assess neurocardiac function as previously described [16]. Briefly, the monitoring of the heart's actions will be performed *via* a three-channel ECG (MLA2503, ADInstruments, Castle Hill, Australia). For this purpose, three self-adhesive ECG electrodes will be fixed to the upper outer left and right pectoral regions as well as the left lower leg close to the ankle. The analogue signals will be pre-amplified with a digital signal amplifier and artefact filter (Bio Amp® ML132, ADInstruments, Castle Hill, Australia) and digitized with the analogue-to-digital converter (PowerLab®, ADInstruments, Castle Hill, Australia). The generated ECG curve will then be displayed using the analysis software (Chart® 5, ADInstruments, Castle Hill, Australia). The baseline HRV measurement will be performed under resting conditions. The subject will be asked to breath with a usual pace and will be resting for at least 3 min prior to the first section of the seminar. During the seminar, HRV will be continuously monitored. The finger electrode will be placed on the index finger of a subject’s non-dominant hand. The HRV will be assessed *via* Standard Deviation of all NN intervals [intervals between normal R-peaks] (SDNN), Root Mean Square of Successive Differences (RMSSD) and Coefficient of Variation of NN Intervals (CVNN). In addition, spectral analysis will be carried out to quantify very low frequency (VLF), low frequency (LF), high frequency (HF), and the sum of all spectra (TP, total power). HRV analysis will be performed separately for phases of student–teacher interaction, post-interaction deep breathing and interim phases of passive seminar attendance.

#### Sudomotor Autonomic Function Assessment: Sympathetic Skin Response (SSR)

Sympathetic skin response will be evaluated to assess sudomotor autonomic function as previously described [17].

Briefly, the SSR will be measured as change in electrical skin conductance (ESC) following sudden deep inspiration using GSR finger electrodes (MLT118F, ADInstruments, Castle Hill, Australia) at the middle phalanges of the third and fourth fingers of the non-dominant hand during three deep inspirations. Before each measurement of skin electrical conductivity, the measurement system will be calibrated and set to zero. The electrical amplifier (GSR Amp® FE116, ADInstruments, Castle Hill, Australia) to which the electrodes are connected generates a low electrical voltage (22 mV). The current flowing through the skin and the applied electrodes are pre-amplified as an electrical signal by the GSR Amp® FE116 and then transmitted to the PowerLab 4/26® instrument (ADInstruments, Castle Hill, Australia), which converts the acquired analogue skin conductance signals into digital data and allows the subsequent curve display and storage of the data by the LabChart Pro® software package (ADInstruments, Castle Hill, Australia). The subject will perform three sudden deep inspirations at baseline, after each interaction with the instructor during both types of seminars and at the end of class. The SSR value will be computed as the maximum increase in amplitude in ESC after deep inspiration: *SSR = ESC post-inspiration (ESCmax) - ESC Inspiration-Onset (ESCmin)*.

### Neurovascular autonomic function assessment: Laser doppler flowmetry (LDF)

Assessment of dynamic vasoconstriction and vasodilatation of microcirculation following sympathetic stimulation will be performed to evaluate neurovascular autonomic function as previously described [18]. Briefly, assessment will be undertaken using laser Doppler surface probes (INL191 Blood FlowMeter LCC, ADInstruments, Castle Hill, Australia) following deep inspiration to stimulate a vasoconstrictory sympathetic response. The LDF technique utilizes a monochromatic coherent laser source on a fixed cutaneous region. The LDF probe will be attached at the middle phalanx of the fourth finger of the non-dominant hand with a circular strip. Processing and computation of the distribution of frequencies of backscattered light enables an approximation of small vessel blood perfusion. The electrical signal from the LDF probe will be transmitted to the PowerLab 4/26® analogue-to-digital converter (ADInstruments, Castle Hill, Australia), which generates the digitised representation of the microcirculation of the skin. Vasoconstrictory response (VCR) as well as the time required (sec) for the vessel to develop 50% constriction or 50% dilation using Δt50%down and Δt50%up values will be computed [18].

### Pupilomotor autonomic function assessment: Pupil light reflex

Controlled induction of the pupil light reflex can be utilized to evaluate function of both branches of the autonomic nervous system, that is, the sympathetic and the parasympathetic system. While pupil constriction, for example, following a light stimulus, is mediated by contraction of iris sphincter muscle under parasympathetic control, pupil dilation, for example, following arousal or the fight-or-flight response, is induced by the sympathetic dilator muscle. Therefore, pupillary diameters as well as homoeostasis of contraction and relaxation of iris muscles reflecting parasympathetic-sympathetic balances. Measurement of the pupil response to a light stimulus, also referred to as the pupil light reflex, allows separate evaluation of the sudden constriction phase and the subsequent phases of re-dilatation. Dynamic assessment of pupil diameter during these phases allows characterization of parasympathetic and sympathetic responsiveness. Moreover, the reflex is modulated by cognitive factors, including attention, processing of visual input as well as awareness making this test particularly valuable in the comparison of learning methods [19]. Therefore, an automated neuro-optical examination consisting of the dynamic assessment of pupil size and pupil reactivity will be performed using a pupillometer (NeurOptics®NPi®-200 Pupillometer, NeurOptics, Inc., CA, USA) with a sampling frequency 32 Hz and accuracy 0.1 mm as previously described [20]. The pupillary margins will be detected in an automated fashion during infra-red illumination. Margins will be tracked during a five-second period of continuous recording following application of a 154 ms light stimulus at an intensity of 180 μW to induce the pupillary light reflex. Resting pupil diameter, pupil light reflex amplitude and latency as well as recovery time and neurological pupil index (NPi) will be computed. The NPi integrates a series of dynamic pupillary variables including size, latency, constriction velocity and dilation velocity into an algorithm to characterize pupillary responsiveness.

### Experimental seminar

One experimental seminar will be held in two consecutive phases (sessions) comprising a phase of face-to-face on-site teaching and a phase video conference based real-time teaching. Each session will last 40 min with a 10-minute break in-between. Prior to the seminar, participating medical students will be informed about objectives during the seminar by the instructor. A multiple-choice test will be undertaken prior and after each seminar session to assess learning progress with respect to the content of the seminar. The content taught will be selected according to the medical curriculum of the participant’s academic year. It will not be redundant to their previous teaching. The learning objectives/goals of the seminar will be mainly focussed on comprehension, which may include background, integration, and solution. The experimental seminars will be conducted throughout the semester. The lectures included in the study were previously involved in designing multiple-choice questions for student exams and have multiple years of teaching experience. However, it cannot be excluded that students had attended previous classes of the instructors delivering the experimental seminars.

#### Learning progress test

Three qualified lecturers, who either hold a professorship or doctorate degree and have multiple years of teaching experience, will provide 10 multiple-choice questions according to seminar’s content including knowledge, problem-based and composite questions. Each multiple-choice question will have five different options with only one correct option. For each seminar session, five identical questions will be handed out to the participating subjects as pre- and post-tests. These tests will be given to students before and after each seminar session. The achieved score after each seminar session will be compared with the score achieved before the seminar begins to reflect the learning success.

### Assessment of emotions

The Profile of Mood States (POMS) questionnaire is a standard validated psychological rating scale used to determine mood states. Altogether, 35 items of emotions and feelings are recorded at baseline and after each section of a seminar using the POMS as previously described [15]. Briefly, the subject will grade their specific emotions and feelings from “0 as not at all”, “1 as a little bit”, “2 as moderate”, “3 as quite a lot”, and “4 as extremely”. All emotions scores will be calculated to indicate Total Mood Disturbance (TMD). Absolute values and percent change in TMD from baseline will be calculated at each time point of measurement.

### Statistical analysis plan

The primary endpoint constitutes a group difference in Standard Deviation of all NN intervals (SDNN). Further endpoints encompass a change of remainder functional parameters of the ANS. The study is conceptualized as a pilot study due to lack of preliminary work. Because of the exploratory nature of this study and absent preliminary research using the same assessment techniques we did not undertake a formal sample size calculation. We intend to allocate 48 subjects (*n* = 48) as targeted sample size.

All statistical calculations will be performed using GraphPad Inplot, ISI software and PRISM Version 5.01 (Graphpad Holdings, LLC, CA, USA). Normality will be assessed by descriptive and analytic methods (Shapiro–Wilk test). Under crossover conditions randomization with a two-period, two-treatment design (2 × 2 design) will be performed. In addition, carryover effects between treatments and periods will be tested using two-way repeated measures ANOVA (mixed model). A two-sided *p* > .05 indicates no carryover effects due to sequencing order. Measured values at each treatment period will then be normalized by mean measured values at baseline. Data include HRV parameters (RMSSD, SDNN, VLF, HF, LF, LF/HF, total power), sudomotor parameters (SCL, SSR), neurovascular function parameters (Δt50%down, Δt50%up, VCR), and pupil dynamics (resting pupil diameter, pupil light reflex amplitude and latency, recovery time, NPi). Between-group differences (baseline vs face-to-face vs videoconference) will be determined using one-way analysis of variance ANOVA followed by Student–Newman–Keul test. Not normally distributed data will be analysed either after log-transformation or *via* non-parametric Friedman two-way ANOVA approach. The significance level is set to alpha = 0.05 and two-sided *p* < .05 is considered significant.

### Data management

The personal data collected in this study will be treated confidentially in accordance with data law, protected from access by unauthorised third parties and may only be published by the executing institutions in anonymized form after the subjects have given their written consent. Anonymity is guaranteed by assigning a student number. Personal data are encrypted in the test forms; only subject numbers are noted in them. Recorded data from measurements will be encrypted as well and kept with labelled subject number. This document will be kept separately from personal data.

### Informed consent

The investigator will obtain informed consent of a subject prior to any study-related procedure. The study protocol will be explained in detail to the subjects by the investigators. In addition, each subject will receive detailed study information in writing. After all questions have been answered, the subject will sign the written informed consent form. One original copy of the written informed consent will be kept in the study centre and a second original copy will be handed out to the subject.

### Limitations and strengths

This study is limited by its pilot nature. However, the detailed autonomic nervous system phenotyping as well as the randomised crossover design ensure high internal validity. Moreover, the results of this study may help to elucidate how the digital transformation of learning in medicine affects psychophysiological health and form a basis for research in large populations of medical learners to identify most effective learning strategies that utilize digital communication and learning tools. Since this is an exploratory pilot study, we will not undertake any validation study prior to the use of the learning progress test. The test will be designed by the lecturers participating in our study based on their expertise in the field. However, the crossover design of our study will ensure comparability of cohorts as each subject will be their own control. This will also minimize inter-individual variability as well as confounding by factors that affect the physiologic and learning responses of the learners to achieve high internal validity.

## Data Availability

N/A (Study Characteristics, no data reported)
